# Predictive Toxicology of cobalt ferrite nanoparticles: comparative in-vitro study of different cellular models using methods of *knowledge discovery from data*

**DOI:** 10.1186/1743-8977-10-32

**Published:** 2013-07-29

**Authors:** Limor Horev-Azaria, Giovanni Baldi, Delila Beno, Daniel Bonacchi, Ute Golla-Schindler, James C Kirkpatrick, Susanne Kolle, Robert Landsiedel, Oded Maimon, Patrice N Marche, Jessica Ponti, Roni Romano, François Rossi, Dieter Sommer, Chiara Uboldi, Ronald E Unger, Christian Villiers, Rafi Korenstein

**Affiliations:** 1 Department of Physiology and Pharmacology, Faculty of Medicine and the Marian Gertner Institute for Medical Nanosystems, Tel Aviv University, Tel-Aviv 69978, Israel; 2 CERICOL, Colorobbia, Via Pietramarina 123, 50053, SoVigliana-Vinci, Firenze, Italy; 3 Institut fuer Mineralogie, Universitaet Muenster, Muenster, Germany; 4 Institute of Pathology - REPAIR Lab, University Medical Center of the Johannes Gutenberg University Mainz, Langenbeckstrasse 1, 55101, Mainz, Germany; 5 Experimental Toxicology and Ecology, BASF SE, GV/TB - Z570 67056, Ludwigshafen, Germany; 6 Department of Industrial Engineering, Faculty of Engineering, Tel Aviv University, Tel Aviv 69978, Israel; 7 Joseph Fourier, U823 Analytical Immunology of Chronic Pathologies Institut Albert Bonniot, INSERM & University Grenoble, BP170, 38042, Grenoble, France; 8 European Commission, Joint Research Centre, Institute of Health and Consumer Protection, Nanobiosciences Unit, via E. Fermi, 2749, 21027, Ispra (VA), Italy

**Keywords:** Nanotoxicology, Cobalt-ferrite nanoparticles, Comparative cytotoxicity, Data mining

## Abstract

**Background:**

Cobalt-ferrite nanoparticles (Co-Fe NPs) are attractive for nanotechnology-based therapies. Thus, exploring their effect on viability of seven different cell lines representing different organs of the human body is highly important.

**Methods:**

The toxicological effects of Co-Fe NPs were studied by in-vitro exposure of A549 and NCIH441 cell-lines (lung), precision-cut lung slices from rat, HepG2 cell-line (liver), MDCK cell-line (kidney), Caco-2 TC7 cell-line (intestine), TK6 (lymphoblasts) and primary mouse dendritic-cells. Toxicity was examined following exposure to Co-Fe NPs in the concentration range of 0.05 -1.2 mM for 24 and 72 h, using Alamar blue, MTT and neutral red assays. Changes in oxidative stress were determined by a dichlorodihydrofluorescein diacetate based assay. Data analysis and predictive modeling of the obtained data sets were executed by employing methods of Knowledge Discovery from Data with emphasis on a decision tree model (J48).

**Results:**

Different dose–response curves of cell viability were obtained for each of the seven cell lines upon exposure to Co-Fe NPs. Increase of oxidative stress was induced by Co-Fe NPs and found to be dependent on the cell type. A high linear correlation (R^2^=0.97) was found between the toxicity of Co-Fe NPs and the extent of ROS generation following their exposure to Co-Fe NPs. The algorithm we applied to model the observed toxicity belongs to a type of supervised classifier. The decision tree model yielded the following order with decrease of the ranking parameter: NP concentrations (as the most influencing parameter), cell type (possessing the following hierarchy of cell sensitivity towards viability decrease: TK6 > Lung slices > NCIH441 > Caco-2 = MDCK > A549 > HepG2 = Dendritic) and time of exposure, where the highest-ranking parameter (NP concentration) provides the highest information gain with respect to toxicity. The validity of the chosen decision tree model J48 was established by yielding a higher accuracy than that of the well-known “naive bayes” classifier.

**Conclusions:**

The observed correlation between the oxidative stress, caused by the presence of the Co-Fe NPs, with the hierarchy of sensitivity of the different cell types towards toxicity, suggests that oxidative stress is one possible mechanism for the toxicity of Co-Fe NPs.

## Background

Magnetic nanoparticles (NPs) possess unique properties which can be applied in nanomedicine: they address targets such as cellular therapy, tissue repair, nanobiosensors, drug delivery, magnetic resonance imaging and magnetic fluid hyperthermia. All these applications require high magnetization values of NPs and size of less than 100 nm with uniform physical and chemical properties [1]. Additionally, it is essential to understand the biological fate and potential toxicity of magnetic NPs for their successful application in nanomedicine [2]. Over the years, Iron oxide, especially magnetite (Fe_3_O_4_), was the most investigated magnetic NP. In the last decade it became easier to synthesize new and more effective types of magnetic NPs [3]. Cobalt- ferrite (Co-Fe) NPs which belong to the crystal family of spinel ferrites (MFe_2_O_4_), posses larger magnetic anisotropy than other ferrites (e.g. magnetite) making them more attractive for nanotechnology based therapies [3,4].

The toxicity of Co-Fe NPs was explored in a number of studies. A significant decrease in cytokinesis-blocked proliferation index and increase in the frequency of micronucleated binucleated lymphocytes were shown when employing 5.6 nm Co-Fe NPs [5]. However, coating the surface of these NPs led to a 4-fold reduced level of toxicity [5]. Similarly, toxicological study of silica-coated Co-Fe possessing a silica shell of 50 nm thickness, revealed that although the particles were found in the mice’s brain, no significant changes in the hematological and clinical biochemistry tests were found [6]. Another study investigated the embryotoxicity of Co-Fe NPs (17 ± 3 nm) through an embryonic stem-cell test which show differentiation into cardiomyocytes. The obtained ID_50_ for the inhibition of differentiation classified Co-Fe NPs coated with gold and silanes as non-embryotoxic. However, Co-Fe NPs coated only with silanes were found to be weakly embryotoxic, but less embryotoxic than the cobalt ferrite salt (CoFe_2_O_4_) [7]. Obviously, further toxicological work should proceed with the aim to achieve a larger toxicological data-base to enable the prediction of toxicology by *in-silico* approaches [8-10].

Predictive toxicology is based on the development of algorithms that are capable of predicting toxic effects (the output) from chemical and biological information (the input) [11,12]. *Knowledge Discovery from Data* (KDD) is the process of identifying valid, novel, useful and understandable patterns from large or complex datasets. KDD can also be applied to small data sets where new insights can be inferred, as is the case of this study. The core of the KDD process is Data Mining (DM), involving the inferring of algorithms that explore the data, develop models and discover significant patterns [13].

This manuscript explores the toxicological effects of Co-Fe (CoFe_2_O_4_) NPs on viability of cells, representing the different organs of the human body, for expanding the toxicological knowledge in the future use of biomedical applications. Dose–response curves were carried out in the concentration range of 0.05 -1.2 mM employing MTT, NR and Alamar blue as endpoint assays following exposures for 24 and 72 h. The cell viability experiments were complemented by determining NP-induced changes of oxidative stress in five of the cell lines. Finally, we applied KDD and DM to data, gathered in the experimental studies, towards the formation of a predictive model of NP toxicity. The predictive modeling was applied by carrying out training and validation through an iterative process when applying the KDD approach. The model predicts the relative hierarchy of the variables studied in the viability tests consisting of concentration, cell type and duration of exposure. A similar approach has been recently reported in a study of the toxicity of Co-NPs and Co-ion [14]. The novelty of the presented model is in its multi-dimensional perspective that cannot be achieved by traditional visual examination of two or even three dimensional plots, as done in most previous toxicological studies.

## Results

The toxicological effects of Co-Fe NPs were examined using seven different cell lines and precision-cut lung slices. Since penetration of NPs into the human body proceeds principally through inhalation or orally, whereas penetration through healthy skin is negligible [15], we have chosen cell lines representing lung (A549 and NCIH441 cell lines) and intestine (Caco-2/TC7 cell line) as the primary sites of interaction. In order to bridge the gap between lung cells and the lung organ, we also examined rat precision-cut lung slices. Liver (HepG2 cell line), kidney (MDCK cell line) and the immunological system (primary murine dendritic cells and a human B-lymphocyte cell line - TK6) have been selected as the secondary major sites of interaction following the penetration of NPs into the blood circulation. Dose–response curves of 0.1-1.2 mM Co-Fe NPs (or 23.5 - 282 μg/ml) were examined employing MTT, neutral red (NR) and Alamar blue as endpoint assays. In addition, ROS levels were explored in five of the cell lines. Data analysis and modeling of the obtained data sets, for the toxicological dose - response curves for Co-Fe NPs were carried out using the approach of *Knowledge Discovery from Data* (KDD).

### Cytotoxic effect of Co-Fe NPs on cells

#### *Cytotoxic effect of Co-Fe NPs on Caco-2 cells*

**Figure 1:**
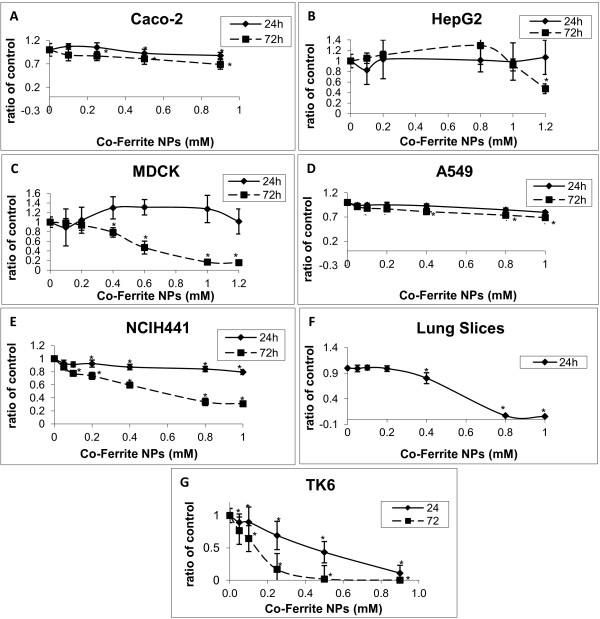
**Effect of Co-Fe NPs on viability assayed by MTT.** Cytotoxicity was examined in different cultured cells: **(A)** Caco-2, **(B)** HepG2, **(C)** MDCK, **(D)** A459, **(E)** NCIH441, **(G)** TK6 and in lung slices **(F)**. Cells were treated with 0 – 1.2 mM Co-Fe NPs for 24 and 72 h. Cell viability was assessed by MTT assays and results are presented as ratio of control (untreated group). Mean ± SD of three independent experiments performed in triplicate. * Significant differences of each point from the control at P<0.05.

**Figure 2:**
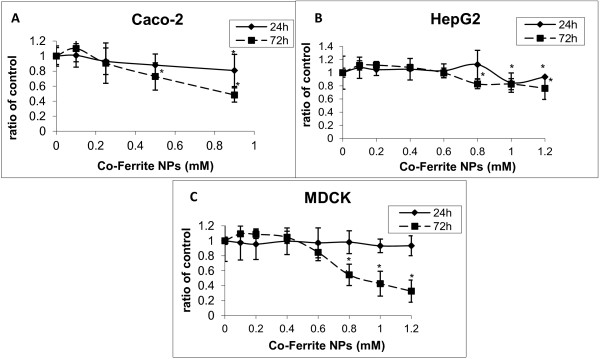
**Effect of Co-Fe NPs on viability assayed by NR.** Cytotoxicity was examined in different cultured cells: **(A)** Caco-2, HepG2 **(B)** and **(C)** MDCK. Cells were treated with 0 – 1.2 mM Co-Fe NPs for 24 and 72 h. Cell Viability was assessed by NR assays and results are presented as ratio of control (untreated group). Mean ± SD of three independent experiments performed in triplicate. * Significant differences of each point from the control at P<0.05.

**Figure 3:**
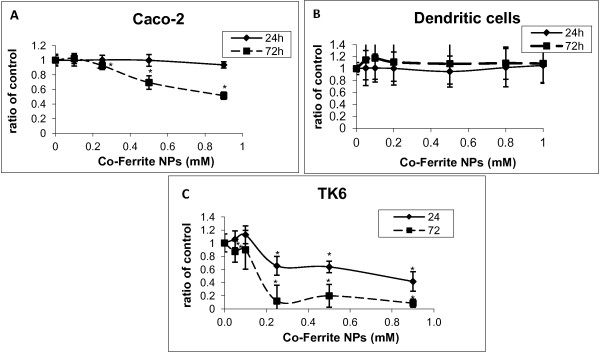
**Effect of Co-Fe NPs on viability assayed by Alamar blue.** Cytotoxicity was examined in different cultured cells: **(A)** Caco-2, **(B)** primary dendritic Cells and **(C)** TK6 were treated with 0 – 1 mM Co-Fe NPs for 24 and 72 h on all different cell lines. Cell Viability was assessed by Alamar blue assays and results are presented as ratio of control (untreated group). Mean ± SD of three independent experiments performed in triplicate. * Significant differences of each point from the control at P<0.05.

Caco-2 TC7 cells, incubated with Co-Fe NPs (0.1-1 mM) for 24 and 72 h, were analyzed employing three different tests for cell viability. These tests, consisting of MTT, NR and Alamar blue assays (Figures 1, 2 and 3, respectively), show that after 24 h Co-Fe NPs do not significantly influence cell viability of Caco-2 cells, however 72 h incubation with Co-Fe NPs causes a significant decrease in viability, especially at concentration of 1 mM, possessing an average EC_50_ of 0.86 ±0.02 mM. This decrease is shown by the NR and Alamar blue assays and less by the MTT assay. When comparing the dose–response curves of the three assays, it turns out that the most sensitive one is Alamer blue. This assay (Figure 3) shows that Co-Fe NPs at a concentration of 0.5 mM induce ~25% decrease in viability while the highest concentration of 1 mM demonstrates viability decrease of 50%.

#### *Cytotoxic effect of Co-Fe NPs on HepG2 cells*

HepG2 cells, incubated with Co-Fe NPs (0.1-1.2 mM) for 24 and 72 h, were analyzed employing two different methods for viability; MTT and NR assays (Figures 1 and 2 respectively). It is shown that incubation with Co-Fe NPs for 24 h does not cause any significant change in cell viability. Incubation with Co-Fe NPs for 72 h reduces cell viability especially at the higher concentration range (1.2 mM). The viability decrease is 15% according to the NR assay and 50% using the MTT assay.

#### *Cytotoxic effect of Co-Fe NPs on MDCK cells*

MDCK cells were incubated with Co-Fe NPs (0.1-1.2 mM) for 24 and 72 h, after which the cells were analyzed by MTT and NR assays (Figures 1 and 2, respectively). The incubation with Co-Fe NPs for 24 h did not result in any significant change in cell viability. However, incubation with Co-Fe NPs for 72 h reduced cell viability significantly (50% reduction) at 0.6 mM and 0.8 mM as shown by MTT and NR assays, respectively. Cell viability reaches attenuation of 70-80% reduction at concentration of 1.2 mM Co-Fe NPs. The average EC_50_ is 0.74 ± 0.19 mM for 72h of incubation.

#### *Cytotoxic effect of Co-Fe NPs on A549 and NCIH441 cells*

A459 and NCIH441 cells were incubated with Co-Fe NPs (0.05-1 mM) for 24 and 72 h, and analyzed by the MTT assay (Figure 1). The highest concentration of Co-Fe NPs (1 mM) causes only 10% and 30% decrease in A549 cell viability after 24 and 72 h respectively. However, whereas 24 h incubation with NCIH441 cells did not cause any significant toxic effect 72 h incubation with Co-Fe NPs caused 40% and 60% viability decrease at concentrations 0.4 mM and 1 mM respectively, possessing EC_50_ of 0.51 mM for 72h of incubation. Thus, the A549 cell line emerges as a less sensitive cell line than the other cell lines and especially in comparison to the other lung cell line NCIH441 which is highly sensitive.

#### *Cytotoxic effect of Co-Fe NPs on TK6 cells*

TK6 cells were incubated with Co-Fe NPs (0.05-1 mM) for 24 and 72 h, and were analyzed by MTT and Alamar blue assays (Figures 1 and 3 respectively). This cell line of B-lymphocytes seems to have the highest sensitivity for Co-Fe NPs, where already at concentration of 0.25 mM 40% of viability decrease has been found after 24 h incubation; after 72 h incubation 0.25 mM cause a viability decrease of 90%. The average EC_50_ is 0.37 ± 0.07 mM and 0.15 ± 0.03 mM for 24 and 72 h of incubation, respectively.

#### *Cytotoxic effect of Co-Fe NPs on Dendritic cells*

Primary Dendritic cells were incubated with Co-Fe NPs (0.05-1 mM) for 24 and 72 h, and analyzed using the Alamar blue assay (Figure 3). These cells which also belong to the immune system, as TK6, were found to be non-sensitive to Co-Fe NPs. The highest concentration of 1 mM does not cause any decrease in cell viability, either after 24 h incubationor after 72 h incubation.

#### *Cytotoxic effect of Co-Fe NPs on lung slices*

Precision-cut lung slices from rat were incubated with Co-Fe NPs (0.05-1 mM) for 24 h and were analyzed by the WST-1 assay (Figure 1). The viability decrease of about 20% already manifests at 0.4 mM Co-Fe NPs. These lung slices’ sensitivity towards the NPs is demonstrated by the lethal effect of an almost 100% decrease in cell viability at NP concentration of 0.8 mM. The EC_50_ for precision-cut lung slices is 0.54 mM.

### Leaching of cobalt ions from Co-Fe NPs

In order to be able to discriminate between the direct toxicological effect of Co-Fe NPs and their indirect effect due the release of Co-ions arising from the dissolution of the NPs, we determined the extent of release of Co-ions from Co-Fe NPs. The exact percentage of leaching was determined for 1.2 mM Co-Fe NPs (the highest concentration that was used in all toxicological assays). Co-Fe NPs were incubated in DMEM with or without 10% serum for several minutes and for 72 h. The total amount of Co-ions in the medium was determined by ICP-MS. The results for Co-ions leaching from the Co-Fe NPs showed minimal amount of Co-ions released regardless to the time incubation or to the presence of FCS.

### Effect of Co-Fe NPs on ROS level in Caco-2, TK6, A549, MDCK and HepG2 cells

**Figure 4:**
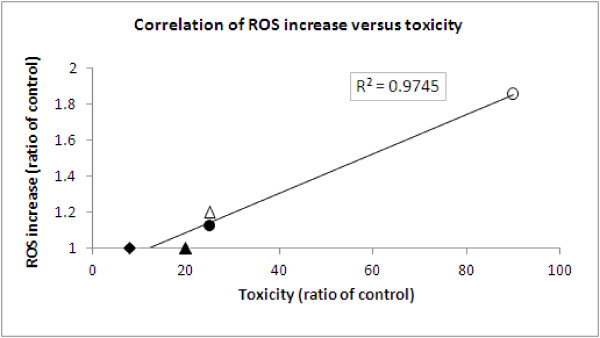
**Correlation between toxicity and ROS level following incubation with Co-Fe NPs.** Correlation between the toxicity (percent of dead cells) after 72 h incubation with 0.5 mM Co-Fe NPs (X axle) versus ROS increase after 1.5 h incubation with 0.5 mM Co-Fe NPs (Y axle); the correlation was fitted linearly yielding y = 0.011X + 0.87; all the results represent the ratio of control cells; the following symbols represent the different cell-lines: open circle – TK6 cells, filled circle – Caco2 cells, open triangle – MDCK cells, filled triangle – A549 cells, filled rhombus – HepG2 cells.

ROS level in the different cell-lines was explored by the DCF method. Table 1 displays an increase in intracellular ROS generation in TK6, Caco2 and MDCK cells, but not in A549 and HepG2 cells after incubation with 0.5 and 0.9 mM Co-Fe NPs. The increase is dose-dependent and in the high concentration is parallel to the positive controls in all three cell-lines; the most sensitive cell-line being TK6 which shows 2.4 fold increase of ROS after incubation with 0.9 mM Co-Fe NPs. However, in A549 cells, which initially seemed very sensitive to oxidative stress (ROS increase by 6.3 folds when exposed to the positive control of TBHP), their ROS level was not affected by Co-Fe NPs at the highest concentration (0.9 mM). Similarly there was no ROS increase in HepG2 cells. In order to assess the relationship between oxidative stress and the averaged toxicity of Co-Fe NPs for the five examined cell lines, we determined the linear correlation between these two parameters. A linear high correlation (R^2^ = 0.97) between the toxicity of Co-Fe NPs (0.5 mM, 72h incubation) and the extent of ROS generation was observed (Figure 4). A lower linear correlation (R^2^ = 0.68) was obtained at a higher concentration (0.9 mM 72 h incubation) of Co-Fe NPs.

**Table 1:** Fold of ROS increase following incubation with Co-Fe NPs

	**TK6**	**MDCK**	**Caco2**	**A549**	**HepG2**
**Co-Fe NPs (0.9mM)**	*2.43±0.56	*1.32±0.26	*1.76±0.15	^**#**^ 1.1±0.11	^**#**^ 0.99±0.16
**Co-Fe NPs (0.5mM)**	*1.86±0.14	1.21±0.21	^**#**^1.13±0.19	^**#**^ 1.01±0.07	^**#**^ 0.97±0.07
**Positive control TBHP**	2.22±0.48	1.46±0.24	1.62±0.09	6.28±0.3	1.75±0.21

Caco2, TK6, MDCK, A549 and HepG2 cells were seeded 48 hours before experiments at concentration of 0.2-0.5x10^6^ cells/well (6 well plate); cells were preloaded with 15 μM H_2_DCF-DA for 1.5 h and then exposure for 24 h with 0.5 mM & 0.9 mM Co-Fe NPs; at the end of incubation time cells were washed, trypsinized (if adherent), washed and the level of the intracellular fluorescent DCF was analyzed by flow cytometry in terms of of the ratio of the geometric mean of exposed cells that of unexposed control cells. The incubation of cells with 0.5 mM TBHP for 45 minutes served as the positive control. The results are the mean of three independent experiments ± standard deviation. * Significant differences of each point from the negative control at P<0.05; # Significant differences of each point from the positive control at P<0.05.

### Patterns discovered from the KDD process

The KDD methodology was applied to the toxicity of Co-Fe NPs in the different cellular models. The modeling was applied by carrying out training and validation through an iterative process when applying the KDD approach. The first KDD goal was to discover rules for determining the toxicity of nanoparticles from the experimental results. The input data set included the consolidated in-vitro experimental results, where each data record has the following attributes: (i) Cell type; (ii) Concentration of Co-Fe NPs; (iii) Exposure time; (iv) The extent of viability decrease. The model predicts the existence or non-existence of toxicity for any combination of the above parameters.

The first phase consisted of data cleansing in order to validate data correctness, thereby avoiding errors for the data-mining process. Data cleansing is part of the methodology of data mining. It means that before applying the data mining algorithms (such as decision tree here) there are necessary, per project, preprocessing stages that look (automatically and by experts) at anomaly and noise in the data, and exclude some data from the later data mining step. The step of data cleansing causes about 4% of the raw data not to be considered due to unreasonable data variation. In the second phase we performed data transformation consisting of: (i) Normalization of results in terms of control and blank results; (ii) Since the results are real numbers while the KDD goal is to determine toxicity (binary definition), we applied an arbitrary discretization rule where a decrease in cell viability, larger than 30% (> EC30) is regarded as a toxic response. This level was chosen taking into account the measurement error. The validity of the threshold chosen was examined by carrying out the same modeling with other discretization thresholds of 25% and 20% for viability decrease.

#### *The description of the model for toxicity*

The algorithm we applied to model the observed toxicity belongs to a type of supervised classifier. We have selected the J48 decision tree classifier since this classifier model can be explained intuitively, in terms of simple if-then rules syntax, without any prior knowledge of data mining techniques (detailed in the methods).

In order to evaluate the classifier, we trained in tenfold cross-validation mode. This was carried out by splitting the data set into ten groups, using nine of the groups for training and the tenth for validation, repeating this process ten times. This method gives robust result for model validation (in essence it does the validation ten times, where each time the test set is randomly chosen). This known ten-fold cross validation method is considered the most robust method for model validation, when machine learning is used. This method gives us a better estimation of the training error (see Methods), since it suffers less from over-fitting.

#### *The sensitivity and validity of the model*

The sensitivity of the model to the choice of toxicity threshold was examined by comparing accuracy and kappa coefficient of the model for different thresholds (we determine a certain threshold for viability decrease to be defined as toxic or non-toxic). The best fit of the model to the data for the threshold values of 30%, 25%, and 20% decrease of viability, was performed: The accuracy of the model, defined as the number of correct predictions divided by the total number of measurements, yielded accuracies of 92.5 % for viability decrease threshold larger than 30% (> EC30), 89% for viability decrease threshold of 25% (> EC25) and 85.2% for viability decrease threshold of 20% (> EC20). The kappa coefficients are 0.74, 0.67 and 0.61 respectively. Since the difference between the three accuracies is significant, the model of viability decrease threshold larger than 30% was chosen, since the measurement error (SD/mean) was in the range of 5-20%. Due to the low level of Co-ion leaching from the NPs, the contribution of the Co-ions dissolution was not considered in the applied model.

The validity of the chosen decision tree model was examined by comparing it to the well-known “naive bayes” classifier. The accuracy of the J48 decision tree model of 92.5% is significantly higher than the accuracy of 84.2 % obtained by the Naive bayes model. The kappa coefficient of the Naïve Bayes model is 0.48, significantly lower than that of the decision tree.

Table 2 shows the performance matrix obtained from the trained decision tree model. Each column of the matrix represents the instances in a predicted class (toxic or nontoxic), while each row represents the instances in an actual class. The values of the confusion matrix enable computing the classifier performance as shown in Table 3. Table 3 also shows the detailed classifier performance by class, i.e. toxic and non-toxic, obtained from the trained decision tree model. The precision for a class is the number of TP (true positives, i.e. the number of items correctly labeled as belonging to the positive class) divided by the total number of elements labeled as belonging to the positive class (i.e. the sum of true positives and FP (false positives, which are items incorrectly labeled as belonging to the class). Recall in this context is defined as the number of true positives divided by the total number of elements that actually belong to the positive class (i.e. the sum of true positives and false negatives, which are items which were not labeled as belonging to the positive class but should have been). The F-Measure is the weighted harmonic mean of precision and recall (*F*_*measure*_*=1/(1/recall+1/precision))*. In the current model true positive ratio for the toxic class is 70% and with F-Measure of above 78%. These are very high performance measures, reassuring us of the results we have stated in this paper.

**Table 2:** Performance matrix

**a = non-toxic**	**b = toxic**	**<−− classified as**
1807	40	a = non-toxic
130	303	b = toxic

The confusion matrix obtained from the trained decision tree model. Each column of the matrix represents the instances in a predicted class (toxic or nontoxic), while each row represents the instances in an actual class.

**Table 3:** Analysis erformance measures by class

**TP Rate**	**FP Rate**	**Precision**	**Recall**	**F-Measure**	**Class**
0.98	0.3	0.933	0.98	0.955	non-toxic
0.7	0.02	0.883	0.7	0.781	toxic

Detailed decision tree classifier performance by class obtained from the trained decision tree model. *TP* (true positives), *FP* (false positives).

## Discussion

The determination of the extent of Co-ions leaching from the NPs due to their dissolution indicates that only a minimal amount of Co-ions leached into the medium, regardless of the incubation-time or the presence of serum proteins. Thus, the highest concentration of 1.2 mM NPs to which the cells were exposed is expected to yield a leaching of 100–110 μM of Co-ions. Such concentration of Co-ions was shown to be non-toxic concentration towards the various cell models examined in this study [14]. Therefore, it is suggested that any toxic effect imposed by the NPs should be attributed only to the direct effect of Co-Fe NPs themselves.

**Figure 5:**
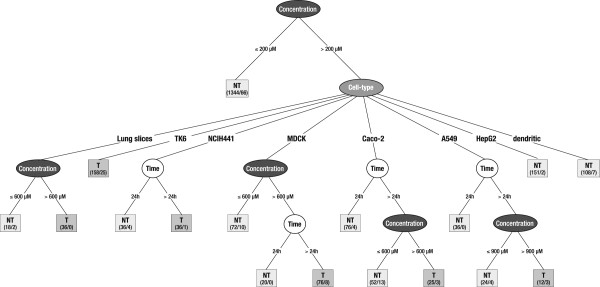
**The decision tree model.** The decision tree model learned from the consolidated results after applying the KDD process described in this work. The decision tree contains different parameters that were used in the data mining procedure: Concentration, Cell-type and time of exposure (24 h or 72 h). The outcome/end of each branch is either nontoxic (NT - the light grey squares) or Toxic (T – the dark grey squares). The numerical results are given below the outcome (NT or T) in the form of N/n_1_, where N represents all data results (NT or T) whereas n_1_ represents the number of data results which do not fulfill the outcome.

The toxicological effects of Co-Fe NPs were examined using seven different cell lines, representing various organs of the human body. The results show different magnitude of decrease in cell viability towards Co-Fe NPs in the different cell lines as shown by the decision tree model in Figure 5. The figure describes the decision tree model obtained from the consolidated results after applying the KDD process (as described in the results). Applying Decision Tree classifier means that the first parameter in the tree root (as depicted in Figure 5) is the one, which is the most important with respect to the observed toxicity (carries most of the information about the toxicity). In our analysis, the concentration feature becomes the highest parameter, as it is the parameter with the maximum information gain, decided by the decision tree algorithm reasoning, with comparison to all other parameters. The concentration parameter is divided according to its values. Each value is in turn a root of a lower decision tree, recursively and with the same logic. The model suggests that incubation with Co-Fe NPs ≤ 200 μM does not cause any toxic effect in all cell types for both incubation times periods. Exposure of cells to concentration above 200 μM leads to differential toxicity between the different cell types; The toxic effect (more than 30% viability decrease) can be seen in lung slices (only 24 h incubation performed), A549, NCIH441, MDCK, TK6 and Caco2 cells, however there is no toxic effect at all concentrations (200–1200 μM) on dendritic cells and HepG2 cells for both 24 h and 72 h incubation times. TK6 cells seem to be the most sensitive cell-line, where Co-Fe-NPs are toxic for TK6 at all concentrations above 200 μM at both incubation times. NCIH441 cells are also sensitive to Co-Fe-NPs above 200 μM but only after 72 h incubation. For both MDCK and Caco2 cells Co-Fe NPs are toxic at concentration higher than 600 μM after 72 h of incubation. Lung slices as well are sensitive to Co-Fe-NPs at concentration higher than 600 μM after 24 h incubation. A549 cells are sensitive to Co-Fe NPs only at concentration of 900 μM and only after 72 h of incubation. Thus the cell type turned out to be the second rank influential parameter. The third and the lowest rank in the model was either the time of exposure (in most cells 24 h incubation caused none or minimal effect) or concentration depending on the cell type. This model suggests the following hierarchy of cell sensitivity towards viability decrease induced by Co-Fe NPs: TK6 > Lung slices > NCIH441 > Caco-2 = MDCK > A549 > HepG2 = Dendritic.

The differential viability response of the various tested cell-lines towards the Co-Fe NPs may be, in principle, attributed to their different capacity of interaction with NPs in terms of either NP adsorption to the cell surface, NP uptake by the cells or both. As the adsorption of Co-Fe NPs to the cell surface is non-specific, one would tend to attribute the observed differential response of the cells to different active endocytic pathways in the various cell-lines. These different pathways can possess dissimilar extent of cargo transport as well as different intracellular fate. Moreover, each cell-line may possess a different repair capacity towards the toxic challenge. It should be pointed out that even cells originating from the same organ (e.g. NCIH441 and A549) may retain different viability response. Out of the three in-vitro models for lung one would tend to assume that the lung-slices, which had the highest sensitivity among the lung models towards Co-Fe NPs, are the more relevant to the in-vivo scenario, when taking into account their higher level of resemblance to lung.

It is intriguing that the two cellular models of the immune system (TK6 and primary dendritic cells (DCs)) turned out to possess the highest and the lowest sensitivity, respectively. DCs are antigen-presenting cells which play a major role in the initiation of the specific immune responses, through their unique ability to stimulate primary T cells. Immature DCs are resident in tissues and epithelial barriers where they act as guardians where they detect phagocyte pathogens. Such activities imply that DCs are equipped to resist to stressing conditions found in inflamed tissues where the amount of ROS produced by macrophages or neutrophils, for example, is very important. Immature DCs produce factors, such as TRANCE (tumor necrosis factor–related activation-induced cytokine) and RANK (receptor activator of NF-κB), that are associated in rescuing of cells from death [16]. Furthermore, DCs express high levels of antioxidant enzymes, such as Mn-SOD and Prx1, conferring the ability to survive in a highly oxidant environment [17]. All these features account for their particular survival ability to stressing conditions such as incubation in the presence of high NPs concentration. The observed much higher sensitivity of lymphocytes vs. dendritic cells is in line with previous studies which also have found a high sensitivity in TK6 cells; An X-irradiation study found that TK6 cells are less efficient in recombinational repair and thus have lower resistance to the toxicity of X-irradiation relative to WTK1 cells, a human B lymphoblast cell line [18]. Another study found that exposure of TK6 to NO, in order to imitate the level in inflamed tissue, caused depletion in reduced glutathione (GSH) level, while the same treatment of activated murine macrophages did not interfere with the GSH homeostasis [19].

The hierarchies obtained from the model are an outcome of the dose–response curves, where the response is an average of viability determined by 2–3 different assay methods (MTT, NR and Alamar blue). This hierarchy is based on a threshold based on EC30 values. It should be stressed that the hierarchy in cell-lines’ sensitivity may depend on the type of NP examined, as well as the conditions of incubation; for example gold NPs were shown to be toxic to A549 cells but not to HepG2 and BHK21 (baby hamster kidney) cells [20].

Oxidative stress, defined as a situation of an imbalance between production of reactive oxygen species (ROS) or reactive nitrogen species (RNS) and antioxidant defenses, is considered to be an important mechanism of NP-induced health effects [21]. It is manifested in the activation of ROS, followed by a pro-inflammatory response and DNA damage leading to cellular apoptosis and mutagenesis [22]. Therefore, it was important to map the response of ROS production of the different cell lines, employed in our study. The obtained results suggest that there is a high linear correlation (R^2^=0.97) between the toxicity of Co-Fe NPs and the extent of ROS generation following their exposure to Co-Fe NPs (Figure 4). In-vitro studies show that NPs generate ROS, deplete endogenous antioxidants, alter mitochondrial function and produce oxidative damage to lipids and DNA [23]. NPs-induced ROS activation promotes defense antioxidant response elements. If damage proceeds, protective systems are succeeded by mitogen-activated protein kinase (MAPK) and NF-κB-activated intracellular signaling, resulting in pro-inflammatory cytokine, chemokine and matrix metalloproteinase (MMP) release leading to apoptosis [22]. Our findings provide evidence to support the notion that the toxicity of Co-Fe NPs is partially due to oxidative stress.

## Conclusions

In the present study we performed toxicological screening of Co-Fe NPs on seven different cell-lines and rat lung slices, showing a high correlation to the observed elevation of the ROS levels. It was validated that any toxic outcome was attributed only to the effect of the Co-Fe NPs themselves (due to a minimal amount of non-toxic Co-ions leaching). We were able to integrate and analyze the toxicological in-vitro data from five different research groups, based on averaged threshold values of dose–response curves of different viability assays, while maintaining data quality control. Data analysis and predictive modeling of the obtained data sets were executed by employing a decision tree model (J48) where training and validation were carried out by an iterative process by applying the *Knowledge Discovery from Data* approach. The modeling of the toxicity data enabled us to obtain a multi-dimensional perspective that cannot be achieved using traditional two or even three dimensional plots. It enabled to determine the hierarchy pattern of the different parameters studied at a threshold of 30% toxicity. Apart from concentration which emerged as the expected parameter of the highest rank, the findings that the second rank parameter is the cell model, as well as the obtained lowest rank for the time of exposure were far from being intuitive. Similar findings were also recently shown for Co-NPs [14]. The presented model agrees with the basic principles of pharmacology and toxicology, yet it organizes their relative hierarchy and thresholds in a clear model based on the attributes of the experimental data. It should be pointed out the present study is restricted in terms of its prediction potential to a specific type of NPs and to the chosen set of the various cell-line. A similar approach, based on decision tree algorithms, can be applied to different types of NPs. A general application of such approach to a much larger database, consisting of different NPs and cell-lines is expected to provide a more general predictive tool for toxicity assessment of NPs. In the last case the physical-chemical properties of the different NPs will be added as additional parameters. Thus, having a very large coherent data-base addressing in-vitro toxicity of different NPs employing large number of diverse cell lines, taking into account the concentration of the particles, duration of exposure and the physical-chemical properties of the NPs, we will be in a position to provide a general model for predicting the toxicity of the NPs.

## Methods

### Materials

Cobalt chloride, (3-[4,5-dimethylthiazol-2-yl]-2,5-diphenyltetrazolium bromide) (MTT), Sodium Dodecyl sulphate (SDS), N,N-Dimethyl formamide, Neutral Red (NR), Tert-butyl hydroperoxide (TBHP), LPS of Escherichia coli 026:B6 were purchased from Sigma (St. Louis, MO, USA). Dichlorodihydrofluorescein diacetate (Carboxy-H_2_DCF-DA) was purchased from Invitrogen (Carlsbad, CA, USA). WST-1 was purchased from Roche (Germany). Alamar blue assay-kit was purchased from Perkin Elmer. GM-CSF is produced by the GM-CSF-transfected J558 cell line (provided by D. Gray, University of Edinburg, UK). Murine recombinant IL-6 and FLT-3 ligand (FLT-3 L) were purchased from Peprotech (Rocky Hill, NJ, USA). The following antibodies, purchased from BD Pharmingen (San Diego, CA, USA) were used: anti-CD40, (3/23), anti-TER-119 (TER119) and anti-Gr1 (RB6-8C5); FITC-conjugated anti-I-Ab/d (25.9.17, Ab), PE-conjugated anti-CD11c (HL3). Female C57BL/6 mice 3 month old were purchased at Charles River (L’Arbresle, France). 8–10 weeks old nulliparous and non-pregnant female Wistar Crl:WI (Han) rats were purchased at Charles River (Germany). Ferric acetate, (Fe(CH_3_COO)_3_) was purchased from the Shepherd Chemical Company (Ohio, USA); Cobalt Acetate (Co(CH_3_COO)_2_.4H_2_O, 23,7% w/w in Co) was purchased from OMG Kokkola Chemicals (Ohio, USA) and diethylene glycol (DEG = O(CH_2_CH_2_OH)_2_) was purchased from Chimica Corona (Fiorano Modenese, Italy); All the chemicals were of reagent grade and were used without any further purification.

### Methods

#### *Synthesis of Co-Fe NPs*

Co-Fe NPs were synthesized employing the following polyol method [24]: cobalt and iron acetates (89,6 and 179,2 mmol, respectively) were solubilized in 645g of Diethylene glycol at 110°C for one hour. The solution was successively heated to 180°C at a heating rate of 2°C/min and then kept at 180°C for three hours. After this growing period the dispersion was air cooled to room temperature and stored. NPs dispersion is stable over a period of two years. Cobalt and iron concentrations of the samples were measured after dissolution of the samples in concentrated nitric acid, the measurement were carried out by solution nebulisation inductively coupled plasma emission spectrometry (ICP-AES, Varian Liberty 51). The final cobalt ferrite concentration was 2.95% (or 2.86%) (w/v); Where the Co/Fe molar ratio being 0.46; 29.5 mg/ml cobalt ferrite NPs (2.95% w/v) corresponds to a concentration of 4.3×10^15^ NPs/ml with a total area of 2.5×10^12^ μm^2^/ml.

#### *Characterization of Co-Fe NPs*

**Figure 6:**
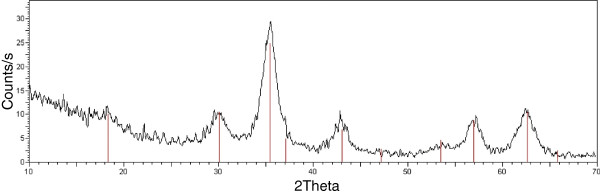
**Characterization of Co-Fe NPs by XRD.** The crystalline structure of Co-Fe NPs was identified from X-Ray diffraction (XRD).

The crystalline structure of the samples was identified from X-Ray diffraction (XRD) patterns recorded in the 2q range 10–70° with a scan step of 0,05°(2q) for 5s on a Philips X’pert pro diffractometer (Cu Ka radiation). The crystallite size was determined from the diffraction peak by using the Scherrer method. The XRD pattern (Figure 6) of the dried sample matches the one expected for the spinel phase characteristic of cobalt ferrite. The average crystallite size was estimated from the X-ray diffraction measurement, by using the Scherrer formula; we obtain 6.10 nm (average of all the peaks). The analysis of the (100) peak gave a diameter of 6.33 nm, that could indicate a non-spherical shape.

**Figure 7:**
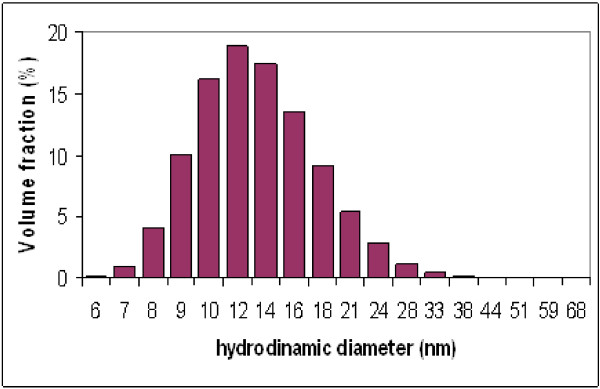
**Size distribution of Co-Fe NPs.** Size distribution of cobalt ferrite NPs by DLS.

DLS measurements were performed on liquids at a concentration of 1 g CoFe2O4/dm^3^ using a Malvern Zetasizer nano-S working with a 633 nm laser beam. The measurement of samples in distilled water (Figure 7) reveals a uniform dispersion of nanoparticles (PDI~0.1) with average diameter of 13.6 nm. DLS measurements over a two years period did not show any significant alteration of the dispersion stability.

To investigate size distribution, morphology, agglomeration and the crystal structure in the range of Ǻ, we use a field emission SEM, the Zeiss 1540 EsB and a conventional JEOL 3010 operating at 297 kV equipped with a LaB6 cathode, post-column Gatan imaging filter and a 1K slow-scan CCD camera.

**Figure 8:**
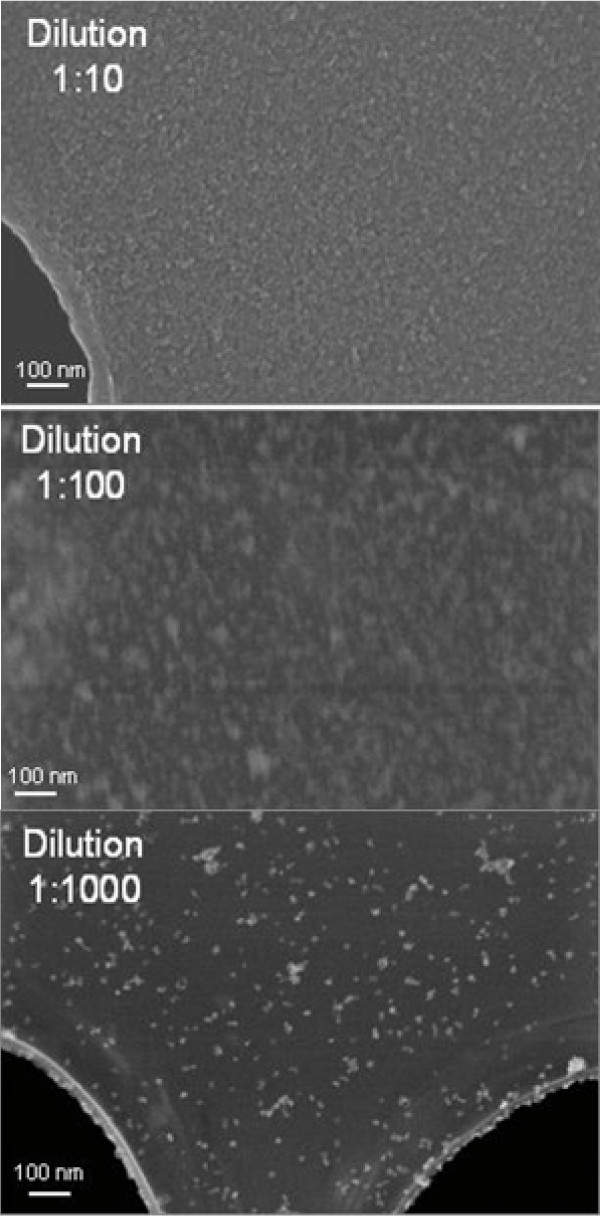
**Characterization of Co-Fe NPs.** SEM images of Cobalt-Ferrite NPs with different dilutions.

The measurements with the SEM were performed using a special STEM detector, which allows studying conventional TEM samples and detecting the reflected signal above the sample as well as the transmitted signal simultaneously. With TEM, the structure information can be obtained by using diffraction patterns that allow distinguishing between amorphous and crystalline areas of the specimen and high- resolution images, which present the lattice fringes of the crystal. Preliminary experiments were performed to determine suitable concentrations of the NPs (Figure 8).

**Figure 9:**
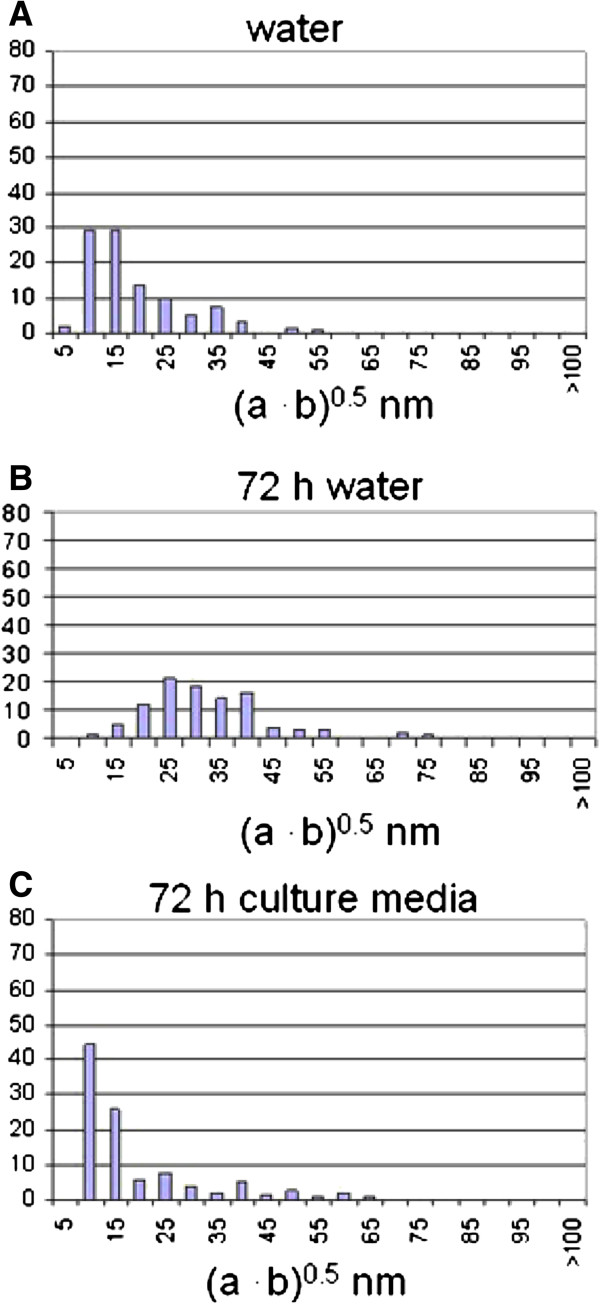
**Size distribution of Co-Fe NPs by SEM.** Size distribution of cobalt ferrite NPs by SEM **(A)** NPs dispersed in water for 0 h, **(B)** 72 h, **(C)** DMEM for 72 h.

Distribution was also examined by SEM; we chose as liquid media DMEM (Dulbecco’s Modified Eagle Medium) as well as water to measure the influence of the physiological medium. All NPs were dispersed in deionized water. The state of dispersion was monitored after some seconds (Figure 9A) or following 72h (Figure 9B) at 37°C, 10% CO_2_, vapor saturation or in cell culture media (DMEM without supplements), for 72 h at 37°C (Figure 9C), 10% CO_2_ under vapor saturation, with a dilution of 1:10. We dropped 20 μl of the selected NP solution on Lacey–carbon or alternatively holey–carbon copper grids laying on filter paper.

**Figure 10:**
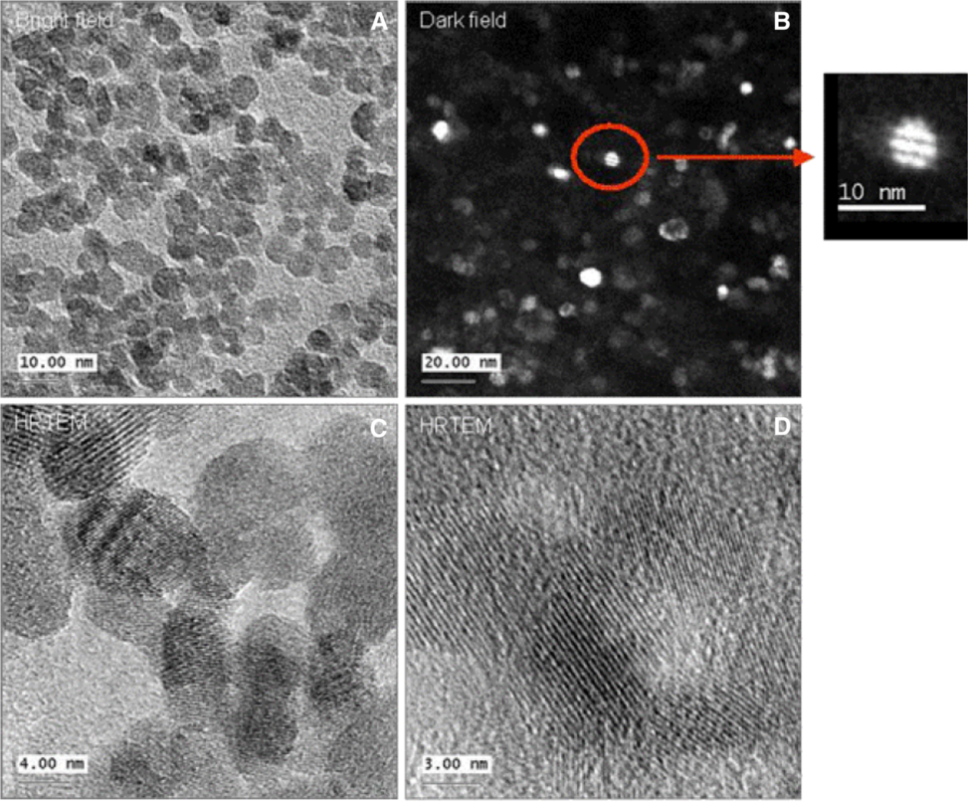
**Characterization of Co-Fe NPs by TEM TEM images of cobalt ferrite NPs. (A)** bright field image, **(B)** dark field image, **(C-D)** high resolution images, inset- magnification of single particle.

To characterize the crystal structure, transmission electron microscopy was used. The zero loss filtered bright field TEM image show a high concentration of NPs (Figure 10A). Dark field imaging has yield one particle with twinning (Figure 10B). High resolution TEM shows mostly single crystals (Figure 10C and D), where the determined lattice spacing was 0.26 nm.

#### *Leaching of cobalt ions from Co-Fe NPs*

The concentration of Co-ions which leached from Co-Fe NPs suspensions was measured by Inductively Coupled Plasma Mass Spectrometry (ICP-MS, Perkin-Elmer SCIEX, Ontario, Canada). We prepared samples diluting the stock suspension (120 mM or 28.14 mg/ml) in complete culture medium and incubated them for 72 h under standard cell culture conditions (37°C, 5% CO_2_, 95% humidity) in the presence and absence of 10% FCS. After incubation, the separation of Co-ions from NPs suspended in culture medium was obtained by filtering the samples using centrifugation (3000 rpm corresponding to 1810 G for 20 minutes) on Millipore-filter membranes of 10kD (Millipore, Italy). In the filtered samples, we measured the amount of Co-ions.

Before the analysis, all the samples were mineralized by adding the appropriate aliquots of concentrated nitric acid (HNO_3_, ultra pure RS, Carlo Erba SpA, Italy), incubated over night at room temperature and microwave digested (MDS-2100, CEM Corp. USA).

#### *Cell Cultures and Rat precision-cut slices*

**A549 and NCIH441 cells** The human alveolar type-II (ATII)-like cell lines A549 (ATCC number CCL-185,) and NCIH441 (ATCC number HTB-174), purchased from LGC-Promochem (Wesel, Germany), were cultured in RPMI 1640 with L-Glutamine (Invitrogen Corporation, Germany) supplemented with 10% (v/v) FCS (Sigma Aldrich, Germany), 10000 U/ml Penicillin and 10000 U/ml Streptomycin (Invitrogen Corporation, Germany). Cells were cultivated in 25 cm^2^ tissue culture flasks and maintained under standard cell culture conditions (5% CO_2_, 95% humidity and 37°C in HERAEUS incubators, Germany). Cells were passaged weekly.

**Caco-2 cells** Caco-2 TC7 cells (a kind gift from Monique Rousset, INSERM U178, Villejuif, France) were cultured in Dulbecco’s Modified Eagle Medium (DMEM), supplemented with L-glutamine (2 mM), 20% FCS and 0.05% PSN solution. All cells were grown in 75 cm^2^ tissue culture flasks (Corning) at 37°C, in a humid atmosphere of 5% CO_2_ in air. Cells were harvested before reaching ~60% confluence by using 0.25% trypsin solution (containing 0.05% EDTA) for 5 min at 37°C. The cells were centrifuged (1 min at 400 g, using Sorvall RT6000D), their solution aspirated and then re-suspended in growth medium. All culture media, antibiotics, trypsin and serum products were purchased from Biological Industries (Beit Haemek, Israel).

**MDCK and HepG2 cells** MDCK and HepG2 cells were supplied by the American Type Culture Collection (ATCC, USA), mycoplasma-free and with source certification. Experimental cultures were prepared from deep-frozen stock vials and always kept in a sub-confluent state. They were maintained in complete culture medium, prepared for MDCK using DMEM high glucose (Invitrogen Corporation, Italy) containing 10% v/v of Fetal Bovine Australian (Invitrogen Corporation, Italy), 2 mM of L-glutamine and 1% v/v of Penicillin/Streptomycin, 10000 U/ml penicillin and 10000 U/ml streptomycin (Gibco, Invitrogen Corporation, Italy). For HepG2, using DMEM high glucose (Invitrogen Corporation, Italy) containing 10% v/v of semi-synthetic FCS II (HYCLONE, CELBIO, Milano, Italy) and 1% v/v of Penicillin/Streptomycin, 10000 U/ml penicillin and 10000 U/ml streptomycin (Gibco, Invitrogen Corporation, Italy). Cell preparations were maintained in standard cell culture conditions (37°C, 5%, CO_2_ and 95% humidity, HERAEUS incubator, Germany) [25].

*TK6*: TK6 cells are human lymphoblastoid cell line (purchased from ATCC). The cells were cultured in RPMI 1640 supplemented with L-glutamine (2 mM), 17% FCS and 1% PSN solution. All cells were grown in 75 cm^2^ tissue culture flasks (Corning) at 37°C, in a humid atmosphere of 5% CO_2_ in air. For each experiment cells were incubated in 6 wells plates (Griener bio-one) at concentration of 0.5 × 10^6^ cells/ well. All culture media, antibiotics, trypsin and serum products were purchased from Biological Industries (Beit Haemek, Israel).

**Dendritic cells** Dendritic cells (DC) were isolated from bone marrow cells of C57BL/6 mice as previously described [26,27]. Briefly, bone marrow cells were recovered by flushing from the femurs. Erythrocytes, TER119 and GR1 positives cells were removed by magnetic cell sorting (Invitrogen, Norway). The remaining negatively sorted cells were resuspended at 5×10^5^ cells/ml in complete Iscove’s modified Dubelcco’s medium (Gibco, Invitrogen, Grand Island, NY, USA) supplemented with 1% of GM-CSF-transfected J558cell line supernatant, 40 ng/ml of mouse recombinant FLT3 L and 5 ng/ml of mouse recombinant IL-6. On day 3, the cellular supernatant was removed and the cells were re-suspended under the same conditions. From day 6 to day 11, IL-6 was removed and FLT3-L was reduced to 20 ng/ml. On day 11, the bone marrow cells are differentiated into DC, ready for the viability tests.

**Precision cut lung slices** Animals (8–10 weeks old nulliparous and non-pregnant female Wistar Crl:WI (Han) rats, (Charles River, Germany) were euthanized with an overdose of pentobarbital-Na. Lung tissue was prepared directly post mortem to conserve the viability of the tissue. Through the trachea the lung was carefully filled in situ with 10 mL/ 200 g body weight pre-warmed 1.5 % agarose – medium solution. The lung was removed and put on ice for 20 minutes, allowing the agarose to polymerize. Lung lobes were separated, placed on wax, and 8 mm tissue cylinders were subsequently prepared. The cylinders were placed into the Krumdieck tissue slicer (Alabama Research and Development, USA) filled with ice cold salt solution (EBSS) and slices with a thickness of approximately 200 μm were prepared. The precision cut lung slices were then washed three times with pre-warmed DMEM/F-12 to completely remove the agarose.

### Cytotoxicity and ROS assays

#### *MTT assay*

The MTT assay is based on the protocol proposed by Dezinot [28]. MTT is a water-soluble tetrazolium salt, which is converted to an insoluble purple formazan by cleavage of the tetrazolium ring by succinate dehydrogenase within the mitochondria. The cell membrane is impermeable to the formazan product and therefore the formazan accumulates in healthy cells. The different cell-lines were seeded in 96 wells at a non-confluent cell density and incubated for 24 h in standard cell culture conditions. After 24 h the medium was removed and the treatment medium was added with different concentrations of Co-ions or Co-NPs as detailed in the results.

After 24 or 72 h of exposure to Co-Fe NPs, 20 μL of MTT solution (5 mg MTT/1mL PBS) was added to each well and the 96 well plates were incubated for 2h at 37°C. 100 μL of lysing buffer (containing SDS and N,N-Dimethyl formamide pH 4.7) was added to each well, and after 1 h cell viability was determined by absorption measurement (λ=560 nm) and the results expressed as the relative viability of cells compared to that of the control cells. The Co-Fe NPs’ background was subtracted from results, where necessary, provided it did not contribute significantly to the total absorbance/fluorescence reading.

#### *WST-1 assay for Rat slices*

After an incubation of the slices for 24 h with different concentrations of Co-Fe NPs, semi-quantitative determinations of the mitochondrial metabolic activity was performed using the water-soluble tetrazolium WST-1 assay. The cell viability was determined by absorption measurement (λ=560 nm) and results were expressed as relative viability of cells normalized to that of the control.

#### *Neutral Red assay*

The neutral red (NR) assay employed is based on the initial protocol described by Borenfreund and Puerner [29], where the accumulation of the neutral red dye in the lysosomes of viable, uninjured cells, related to active transport of the dye, is determined. The different cell-lines were seeded in 96 well plates at a non-confluent cell density and incubated for 24 h under standard cell culture conditions. After 24 h the medium was removed and the treatment medium was added with different concentrations of Co-Fe NPs as detailed in the results. After 24 and 72 h of exposure to Co-Fe NPs, the treatment solution was replaced with 100 μL of filtered NR stock solution (2 mg NR/1 mL) dissolved in appropriate growth medium (1:40) added to each well for 2 h in 37°C, followed by rapid washing with PBS or medium w/o serum. Then, the cells were agitated with 100 μL of a bleaching solution containing 50% v/v ethanol, 49% v/v ultrapure water and 1% v/v acetic acid for 20 minutes. Cell viability was determined by absorbance measurement (λ=544 nm) and results were expressed as relative viability of cells, normalized to that of the control cells. The Co-Fe NPs’ background was subtracted from results, where necessary, provided it did not contribute significantly to the total absorbance/fluorescence reading.

#### *Alamar Blue assay*

The Alamar Blue assay incorporates a fluoremetric or colometric indicator based on detecting metabolic activity. Alamar blue (resazurin) is a nontoxic oxidation-reduction indicator which is reduced by the metabolic activity of the cells reflecting both viable cell number and changes in cellular redox activity [30]. The different cell-lines were seeded in 96 wells at a non-confluent cell density and incubated for 24 h under standard cell culture conditions. After 24 h the medium was removed and cells were exposed to different concentrations of Co-Fe NPs as detailed in the results. After 24 h and 72 h of exposure to Co-Fe NPs, 100 μL of Alamar Blue solution (diluted 1:10 from stock solution) was added to each well and the 96well plates were incubated for 2-3 h in an incubator (37°C). Cell viability was measured in by fluorescence (λ_ex_=485 nm, λ_em_=595 nm) and results were expressed as relative viability of cells compared to that of control cells. The Co-Fe NPs’ background was subtracted from results, where necessary, provided it did not contribute significantly to the total absorbance/fluorescence reading.

#### *Determination of intracellular ROS based on dichlorodihydrofluorescein diacetate (H*_2_*DCF-DA) fluorescent probe*

Dichlorodihydrofluorescein diacetate (H2DCF-DA) (Carboxy-H_2_DCF-DA -Invitrogen D400) was used to determine changes in ROS activity. It permeates the cell membrane and accumulates mostly in the cytosol following deacetylation by esterases to dichlorodihydrofluorescein (DCFH). This non-fluorescent product is converted by ROS into DCF (Ex=488 nm; Em= 525 nm). The different cell-lines were seeded in 6 well plates at a non-confluent cell density (for the adherent cells) and incubated for 24 h under standard cell culture conditions. After 24 h the medium was removed and the treatment medium containing 0.5 or 0.9 mM of Co-Fe NPs was added. After 1.5 h of exposure to Co-Fe NPs, the treatment solution was replaced with Carboxy-H_2_DCF-DA (15 μM) by adding it to the free-serum medium. After incubation for 1.5 hour, cells were washed and the intracellular fluorescence of DCF was measured by flow cytometry (FACSCalibur flow cytometer from Becton Dickinson Company). The optical interference from the Fe-Co NPs in the flow cytometric measurements of the dichlorodihydrofluorescein is unlikely as no changes in the side scattering were observed in the presence and absence of Co-Fe NPs. Results are expressed in terms of Gmean relative to that of control; Exposure to TBHP (tert-butyl hydroperoxide) (0.5 mM) for 0.5 hour was used as a positive control.

The statistical significance of the changes for the different assays of cell viability using specific cell lines was determined by two-tailed Student’s *t* test. Differences of <0.05 were considered statistically significant.

### Methods for *knowledge discovery from data*

*Knowledge Discovery from Data* (KDD) is an automatic, exploratory data analysis and modeling of complex data sets, such as were available during the present research on the toxicological effects of NPs. KDD is an organized process of identifying valid, novel, useful, and understandable patterns from large or complex datasets [13]. Data mining (DM) is the core of the KDD process, involving inferring algorithms that explore the data, develop the model and discover previously unknown patterns. The model is used for understanding phenomena from the data, analysis and prediction. The process of KDD starts with determining the KDD goals, and ends with the discovered knowledge implementation [13]. A brief description of the nine steps of the KDD process is detailed in a previous article [14].

#### *Data mining algorithms*

Supervised learning methods [13] can be applied to predict the toxicity of nanoparticles based on models derived from experimental studies. These methods are used to discover a functional relationship between independent variables (concentration of Co-Fe NP, cell type, exposure time) and a target attribute (toxic or non-toxic), based on a training set, which is a set of input (the independent variables) – output (the target attribute) pairs which hopefully describe the relationship in an unbiased manner.

#### *The decision tree classifier method*

Usually there is a distinction between regression methods whose output is real valued (numeric) and classification method whose output is categorical (nominal or member of an unordered set). In this study we apply the classification methods since we define toxicity in a categorical manner (toxic or not-toxic). The specific method chosen is based on a decision tree classifier. This is a rooted tree (shown in Figure 5) where each of its nodes represents a partition of the samples, according to a function of the input attribute [13]. There are many advantages to using decision tree for modeling: (1) Decision trees are simple to understand and interpret; (2) Decision trees have value even with little hard data, important insights can be generated; (3) Decision trees can model non-linear relationships (4) Decision trees are white box models: if a given result is provided by a model, the explanation for the result is easily replicated. There are several algorithms for generating a decision tree; perhaps the most used one is based on information theory concepts: We look for the attribute with the highest information gain (concentration or exposure time or cell type), and split accordingly, creating a node for each of the possibilities, and repeat this process recursively for each node, with the information gain calculated according to the new conditional distribution. There are two cases where a node isn’t being divided: (1) In all the above instances the target attribute is the same; (2) all the input attributes are the same, thus not allowing a way to distinguish between the different situations; in this case we simply create a leaf that predicts the majority.

#### *The Weka implementation - the J48 decision tree algorithm*

The Weka implementation [31] applied in this work, the J48 decision tree algorithm, is based on the well-known C4.5 algorithm [32]. This implementation starts with large sets of cases belonging to known classes. The cases, described by any mixture of nominal and numeric properties, are inspected for patterns that allow the classes to be reliably discriminated (i.e. toxic or non-toxic). These patterns are then expressed as models, in the form of decision trees or sets of if-then rules that can be used to classify new cases. In order to examine the dependence of the obtained results on the model chosen we compared the outcome of J48 decision tree algorithm with that of Naïve Bayes model. The Naïve Bayes is a classifier that predicts the value of the target attribute, based on probability estimation [31]. The classifier asks to find the target attribute value that maximizes the conditional probability of the target attribute given the input attribute. In general, these tasks are impractical. However, under the assumption of independence of the input attributes (e.g. Co-Fe NPs, concentrations, exposure duration, assay used) for a given target attribute (toxicity), these tasks becomes very easy and efficient, since the calculation of the desired probability is very simple based on Bayes theorem. Finally, as shown in the results the J48 decision tree algorithm found to be with significantly higher accuracy and higher kappa coefficient than the accuracy and kappa coefficient obtained by the Naive bayes model, therefore J48 decision tree algorithm was chosen to be used for our model. We can also define the training error (as described below), which is a common measure for evaluating the model performance: the ratio between the numbers of records wrongly predicted after testing the model on the same training set, and the whole number of records.

#### *Empirical estimation of the training error - cross validation*

The main goal of the models described above is to minimize the training error [13] – the expected rate of wrong prediction (unknown since we don’t know how the distribution of the population space). Cross-validation is one of several approaches to estimating how well the model one has learned from some training data [13] is going to perform on future as-yet-unseen data. Thus cross validation estimates the training error by splitting the known examples into two groups: the training set and the test set. First we use the training set in order to create a classifier and then we test the measure of its misclassification on the test set. This method gives us a better estimation of the training error, since it suffers less from over-fitting.

## Abbreviations

Co-Fe NPs: Cobalt-ferrite nanoparticles; KDD: *Knowledge Discovery from Data*; DM: Data Mining; DCs: Dendritic cells.

## Competing interests

The authors declare that they have no competing interests.

## Authors’ contributions

GB and DB were performed the synthesis and characterization of Co-Fe NPs. UGS and DS carried out the TEM charaterization of Co-Fe NPs. SB and RL carried out the toxicological screening of precision cut lung slices. JCK, CU and REU carried out the toxicological screening of A549 and NCIH441 cells. JP and FR carried out the toxicological screening of HepG2 and MDCK cells and determined the extent of leaching of cobalt ions from Co-Fe NPs. PNM and CV carried out the toxicological screening of the dendritic cells. DB carried out the toxicological screening on Caco-2 and TK6 cells. LHA and RK carried out the oxidative stress assay on all cells, collected and assembled the toxicological raw data for the data analysis and data mining and drafted the manuscript. RR and OM performed the data analysis and predictive modeling of the obtained data sets employing the KDD approach. All authors read and approved the final manuscript.
